# Case report: A novel perspective on the treatment of primary tracheal small cell carcinoma: a patient’s experience with immuno-combined EP therapy and literature review

**DOI:** 10.3389/fimmu.2024.1356268

**Published:** 2024-01-29

**Authors:** Yu Chen, Hongbin Zhu, Danping Wang, Yalan Ye, Jianrong Gao

**Affiliations:** Department of Respiratory and Critical Care Medicine, Chaohu Hospital Affiliated with Anhui Medical University, Chaohu, China

**Keywords:** tracheal small cell carcinoma, immune-combined therapy, etoposide-platinum regimen, adebrelimab, optimal treatment strategy

## Abstract

Tracheal small cell carcinoma (SCC) is a rare malignancy, for which the optimal treatment strategy has yet to be determined. Currently, treatment largely aligns with the therapeutic guidelines established for small cell lung cancer, although numerous unresolved issues remain. This paper details a case study of a patient with Stage IIIB primary tracheal SCC, who was treated with an immune-combined etoposide-platinum(EP) regimen. This treatment offers valuable insights into innovative approaches for managing such malignancies. Furthermore, the study includes a comprehensive literature review to better contextualize the findings. The patient, admitted on May 2, 2023, had been experiencing persistent symptoms of airway discomfort for 15 days. A bronchoscopy performed on May 4 revealed tracheal SCC, classified as T4N2M0, IIIB. Following the CAPSTONE-1 study’s methodology, the patient underwent six cycles of PD-L1(adebrelimab) combined with EP therapy, leading to significant relief of symptoms and the eventual disappearance of the tracheal mass.

## Introduction

Primary tracheal cancer, a relatively uncommon malignant respiratory tumor, represents approximately 0.2% of such neoplasms (1). Its pathological subtypes include squamous cell carcinoma, adenoid cystic carcinoma, among others, with rarer forms like adenosquamous carcinoma, mucinous epidermoid carcinoma, undifferentiated glandular carcinoma, melanoma, and chondrosarcoma (2). In the early stages of the disease, symptoms often lack specificity (3), and some individuals may exhibit manifestations like cough, sputum production, chest tightness, and shortness of breath, often leading to misdiagnosis as other respiratory conditions like chronic obstructive pulmonary disease or bronchitis (4). Diagnosis is definitively established through imaging and histological examinations. As the tumor grows, obstruction of the tracheal lumen may occur, resulting in symptoms of respiratory distress. Involvement of blood vessels may lead to hemoptysis, and when lymph nodes are affected, symptoms such as lymphadenopathy and tenderness may manifest (2). Among these, SCC of the airway is particularly rare, with only a limited number of cases described. The clinical characteristics of tracheal SCC closely resemble those of squamous cell carcinoma of the airway, encompassing factors such as patient age, gender, ethnicity, disease severity, lymph node involvement, and treatment modalities, including surgery and radiation. Notably, patients with tracheal SCC are more likely to receive chemotherapy (5). Some scholars have posited that treatment regimens effective for small cell lung cancer might also be applicable to tracheal SCC (6). However, the optimal therapeutic approach for tracheal SCC continues to be a topic for further investigation. Addressing the need for a more comprehensive understanding of tracheal SCC, this paper reports on a case of airway SCC where significant relief was achieved through the adoption of immunotherapy combined with EP treatment, informed by the CAPSTONE-1 study methodology. methodology, this report includes a thorough review of relevant literature.

## Case presentation

**Figure 1:**
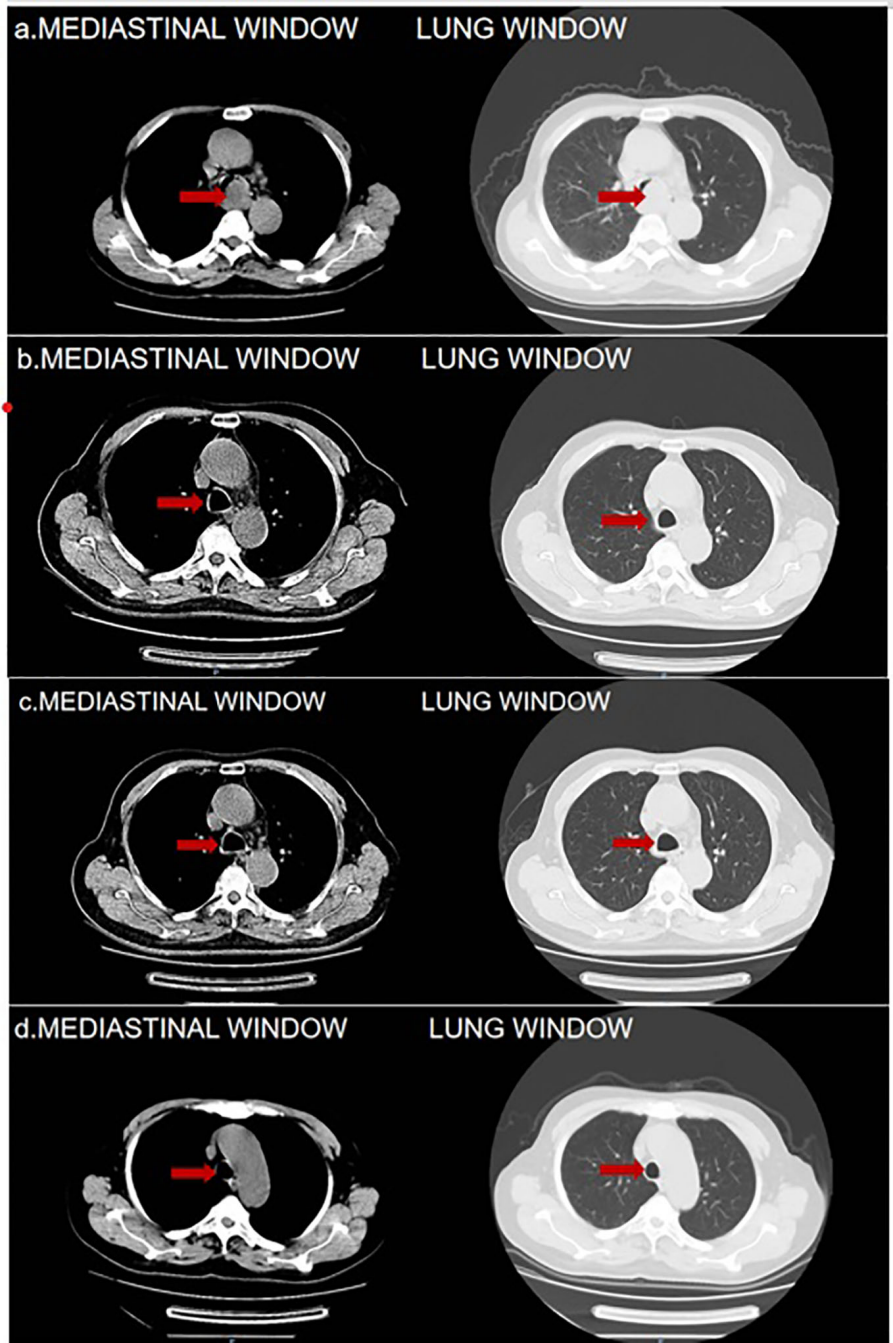
**(A)** 2023-5-2 Pulmonary CT Findings: Tracheal Prominence Occupancy. **(B)** 2023-6-28Pulmonary CT Findings: Significant Reduction in the Mass Below the Tracheal Prominence. **(C, D)** 2023-8-22 and 2023-11-1Pulmonary CT Findings: Absence of Mass Below the Tracheal Prominence in Both. The red arrows are used to indicate the location of tumor.

On May 2, 2023, an 83-year-old male was admitted, reporting a 15-day history of recurrent cough, sputum production, chest tightness, and wheezing. The patient described an exacerbation of these symptoms, characterized by white, viscous sputum, and noted that the chest tightness and wheezing intensified with physical activity. Despite self-medicating (details unspecified), the symptoms showed no significant improvement. Consequently, the patient sought medical care at our outpatient clinic. A pulmonary CT scan conducted on the same day revealed a lesion at the tracheal prominence indicative of malignant tumor (MT), enlarged mediastinal lymph nodes suggestive of lymphatic metastasis, a small nodule in the right lower lobe, and scattered inflammatory lesions in both lungs. The scan also showed signs of bronchiectasis and pulmonary emphysema (**Figure 1A**). The patient’s medical history was notably unremarkable for major illnesses or exposures, and there was no history of substance abuse or familial diseases. Comprehensive examinations conducted on May 3, 2023, including blood tests and scans, yielded normal results, apart from elevated tumor markers (CEA, NSE, and ProGRP).

**Figure 2:**
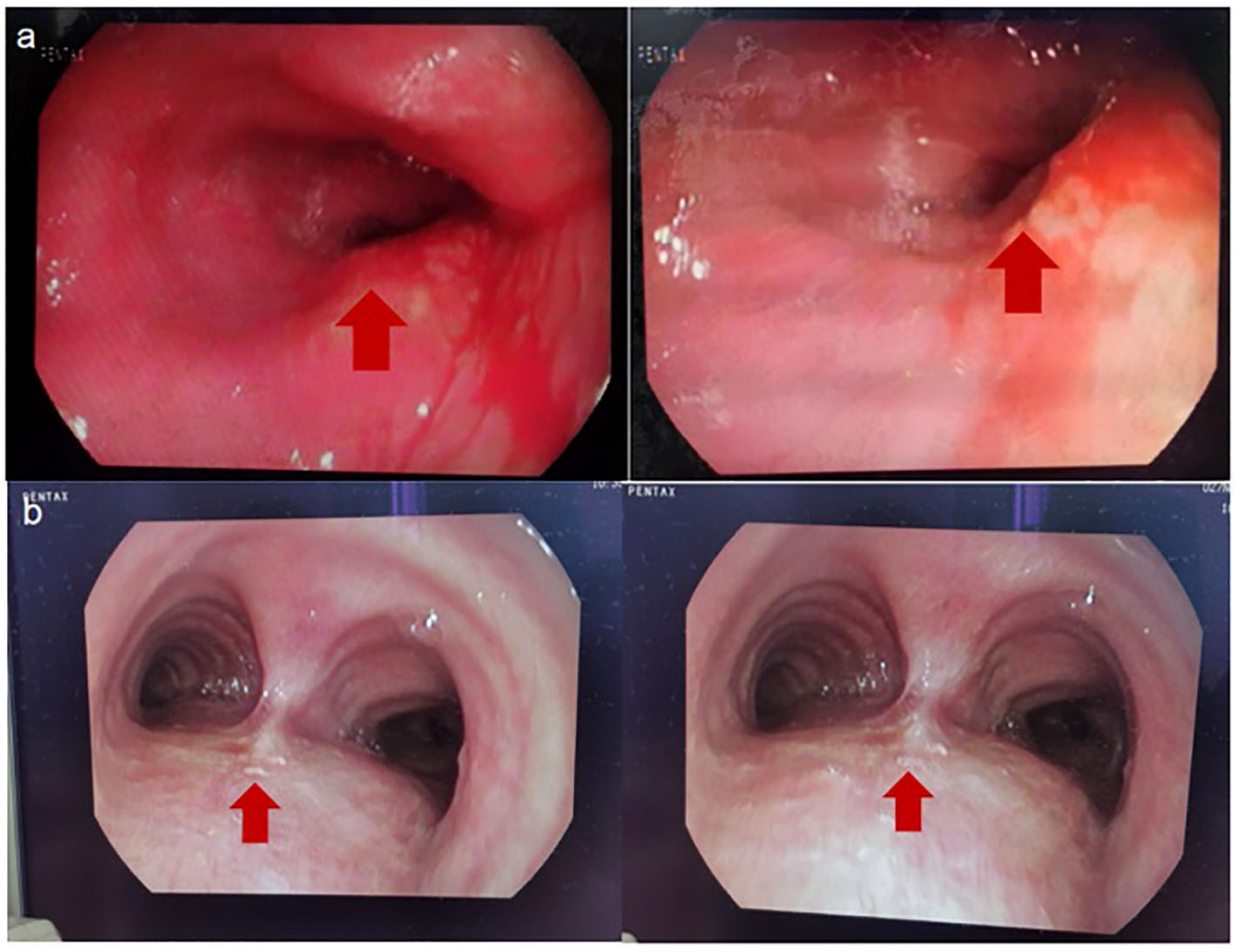
**(A)** 2023-5-5 Fibrobronchoscopy Reveals: Compression of the Tracheal Glottis, Narrowing of the Lumen, and Mucosal Swelling and Congestion. **(B)** 2023-11-2 Fibrobronchoscopy Reveals: The Tracheal Eminence Mass Has Disappeared, Localized with Slight Nodular Protrusions. The red arrows are used to indicate the location of tumor.

**Figure 3:**
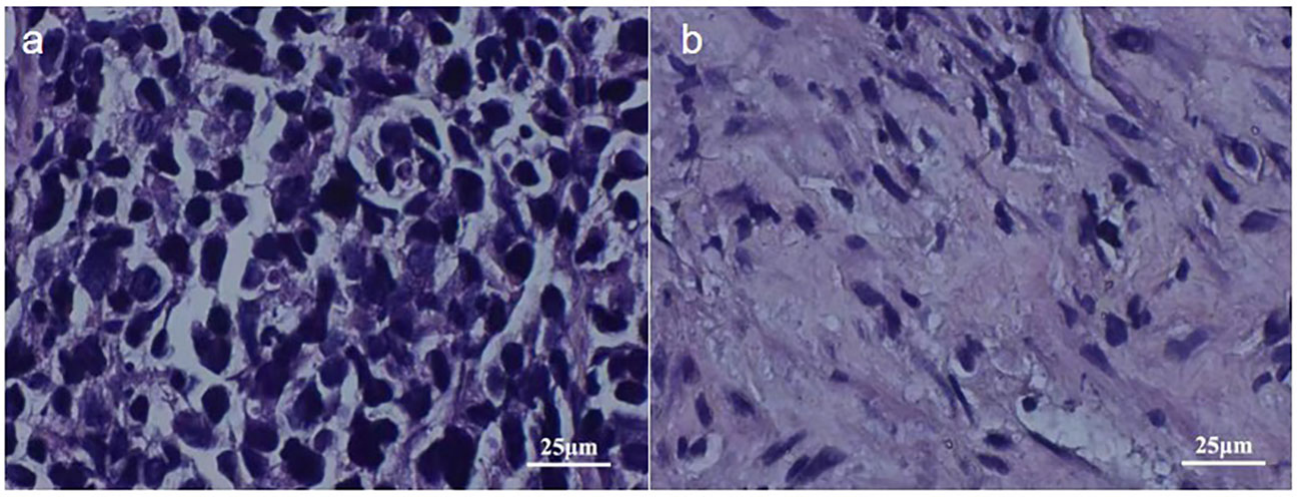
2023-5-5 Pathological Images: Image **(A)** Displays Tumor Tissue Cells Under 400x Magnification, Image **(B)** Presents Cells of Para-cancerous Tissue Under 400x Magnification.

On May 5, 2023, a bronchoscopy identified an extrinsic compressive growth causing tracheal stenosis, with mucosal swelling and an irregular surface, approximately 13 cm below the glottis. The narrowed tracheal lumen measured about 10x5 mm, where a mucosal biopsy was obtained (**Figure 2A**). Histopathological examination and immunohistochemistry combined with diagnosis of tracheal SCC (**Figure 3**), with the tumor tissue expressing CD56+, TTF-1+, NapsinA-, P40-P63-, and LCA-Ki-67 approximately 90% positive. The patient, classified as stage IIIB (T4N2M0) with an ECOG performance status of 1, tolerating the treatment well and exhibiting no significant adverse reactions. Follow-up pulmonary CT on June 28 (**Figure 1B**), August 22 (**Figure 1C**), and November 1, 2023 (**Figure 1D**), demonstrated a marked reduction in the tracheal prominence An electron bronchoscopy on November 2, 2023, revealed slight scarring and some point-like projections at the lower end of the tracheal prominence, without tracheal obstruction (**Figure 2B**).

Additionally, from the initial admission until November 5, 2023, the patient was subjected to regular monitoring encompassing blood routine, liver and kidney function, myocardial enzyme spectrum, and thyroid function tests. During this monitoring period, mild fluctuations were observed in thyroid hormone levels, which ranged between 40-46 mg/L and a slight leukopenia, varying between 1-2 degrees, were noted. Other parameters, including liver and kidney function tests and myocardial enzyme spectrum, consistently indicated no significant abnormalities. Importantly, throughout this period, the patient did not develop any immune-related dermatomyositis.

## Discussion

Primary tracheal tumors are considered rare, with primary SCC of the trachea being notably elusive, and its optimal treatment strategy remains insufficiently explored. Presently, clinical approaches often draw upon the treatment protocols established for small cell lung cancer. Nonetheless, significant inconsistencies are observed in the staging of tracheal malignant tumors compared to lung malignancies, along with variations in diagnostic and therapeutic procedures. To address this, a distinct staging and diagnostic-treatment workflow has been developed, employing the TNM staging system for classification (**Table 1**). This system stratifies cases into four stages based on diverse TNM manifestations (**Table 2**). Despite the availability of this independent staging protocol, ta gap persists due to the lack of large-scale clinical research focused on optimal treatment strategies. As a result, treatment procedures are predominantly based on clinical staging (**Table 3**) (7, 8). The primary treatment approach for these cases typically involves a combination of radiation therapy and chemotherapy; however, the efficacy of various regimen remains to be definitively established. Furthermore, the management of tracheal SCC is characterized by its diversity, potentially influencing the overall treatment effectiveness. Building upon the foundation of TNM staging, this study entails a retrospective analysis of global tracheal SCC cases reported since 2004. The subsequent table (**Table 4**) delineates the current challenges in treatment (4, 6, 9, 10):

**Table 1:** Paolo Macchiarini TNM staging of tracheal malignant tumors.

**Tumor (T) Staging**	Tx	Primary tumor cannot be evaluated
	Tis	Tumor in situ, no invasion
	T1a	<3cm, confined to mucosa
	T1b	≥3cm, confined to mucosa
	T2	Tumor invades cartilage or outer membrane; tumor involvement of the membrane is at least classified as T2, regardless of the depth of invasion
	T3	Tumor invades the trachea or larynx
	T4a	Tumor invades the carina or main bronchus
	T4b	Tumor invades adjacent structures
**Lymph Node (N) Staging**	Nx	Regional lymph nodes cannot be assessed
	N1	Positive regional lymph nodes (N1a <3cm; N1b ≥3cm)
	Upper 1/3	Superior mediastinal lymph nodes; upper tracheal lymph nodes; prevascular and retrotracheal nodes
	Middle 1/3	Upper tracheal lymph nodes; prevascular and retrotracheal nodes; lower tracheal lymph nodes; paratracheal nodes (aortopulmonary window)
	Lower 1/3	Upper tracheal lymph nodes; prevascular and retrotracheal nodes; subaortic nodes (aortopulmonary window)
	N1A	1-3 nodes in upper one-third
	N1B	>3 nodes in upper one-third
	N2	Positive regional lymph nodes
	Upper 1/3	Lower tracheal lymph nodes; subaortic nodes (aortopulmonary window)
	Middle 1/3	Superior mediastinal lymph nodes; subaortic nodes (aortopulmonary window)
	Lower 1/3	Upper tracheal lymph nodes; ligamentum arteriosum
**Metastasis (M) Staging**	Mx	Distant metastasis cannot be evaluated
	M0	No distant metastasis
	M1	Distant metastasis beyond N1 and N2 regions
	M2	Distant metastasis (e.g., in the lungs)

**Table 2:** Paolo Macchiarini TNM staging of tracheal malignant tumors.

Staging	T	N	M
0	Tis	N0	M0
Ia	T1a	N0	M0
Ib	T1b-2	N0	M0
IIa	T1b-2	N1	M0
IIb	T1b-2	N2	M0
IIIa	T3	N0	M0
IIIb	T3	N1-2	M0
IVa	All	N1-2	M1
IVb	All	N1-2	M2

**Table 3:** Diagnostic and therapeutic workflow for malignant tracheal tumors.

Staging	Substage	Objective	Management Strategy
0/I	0/I	Treatment or Observation	Surgical and other local curative treatments or observation
II	IIa/IIb	Cure	Surgical and other local curative treatments, with some adjuvant therapy
IIIa	IIIa/IIIb	Potential cure possibility	Surgical and other local curative treatments for some; majority may require combined systemic therapy and multidisciplinary care
IIIb	IIIb	Potential cure possibility	Multidisciplinary care, surgical and other local curative treatments, and systemic therapy for the majority
IVa	IVa	Potential cure possibility	Multidisciplinary care, systemic therapy for the majority, and some surgical and other local curative treatments
IVb	IVb	Potential cure possibility	Mainly systemic therapy with multidisciplinary care, symptomatic supportive treatment, and palliative care

**Table 4:** Reported cases of tracheal small cell carcinoma in the last 20 years.

**Case No.**	1	2	3	4
**Gender**	Male	Female	Male	Male
**Age (years)**	28	25	73	77
**Discovery Year**	2005	2007	2016	2023
**Symptoms**	Cough, Hoarseness, Swallowing Difficulty	Dyspnea, Hemoptysis	Dyspnea, Hoarseness	Dyspnea
**Treatment Regimen**	Cisplatin and etoposide for 4 cycles; 1.5 Gy irradiation for 30 sessions	Cisplatin for 6 cycles; Conformal radiation therapy with 6 MV photons, total dose of 5600 cGy in 28 fractions over 38 days	6 cycles (etoposide 100 mg/m^2^, cisplatin 80 mg/m^2^, every 3 weeks) + 10 sessions of radiation therapy (3000 cGy)	Cycle 1: carboplatin (AUC = 5mg/mL/min) and etoposide (80 mg/m^2^). Cycle 2 onwards: cisplatin and etoposide (60 mg/m^2^); Cycles 3-4 with added AHF (45 Gray/30 fractions). After complete remission, whole-brain irradiation (25 Gray/10 fractions)
**Primary**	Yes	No	Yes	Yes
**Overall Survival Time(Follow-up time)**	21 months	In the referenced article, the patient's survival time is not mentioned	5 years	In the referenced article, the patient's survival time is not mentioned

In the four cases under consideration, the predominant symptoms included respiratory distress and hoarseness, with some patients also presenting with cough and swallowing difficulties, indicating possible esophageal involvement. Of these cases, three were identified as primary, while one was classified as secondary. All cases were treated with a combination of chemotherapy and radiotherapy. The maximum recorded survival period was 5 years, though the total survival durationfor some cases has not been comprehensively documented. Notably, tracheal SCC is characterized by a wide age range at onset, prolonged overall case duration, scarce reported instances, extremely low incidence rates, and no apparent gender predilection. These cases highlight that the optimal treatment modalities for tracheal SCC necessitate further in-depth investigation. Typically, tracheal SCC presents as an intrabronchial mass, and currently, there are no well-established treatment guidelines. The primary therapeutic strategies are derived from historical treatment experiences and include surgical intervention alone, combined surgery and chemotherapy, or chemotherapy alone (6). Over the past few decades, EP chemotherapy has remained the standard frontline treatment for extensive-stage small cell lung cancer (ES-SCLC), with no significantly superior alternatives yet identified. This regimen typically involves cisplatin and etoposide, administered with or without concurrent radiotherapy. Despite the initial sensitivity of ES-SCLC to EP chemotherapy, resistance development is nearly inevitable, leading to tumor recurrence within six months and an objective response rate of approximately 50-60%. Notably, over the past two decades, no significant breakthrough in medical interventions have been achieved, and patient outcomes have not demonstrated marked improvement (11). In contrast, monotherapy with chemotherapy tends to yields less favorable results, and the addition of radiotherapy can introduce unpredictable risks (12). The optimal therapeutic approach and overall efficacy for tracheal SCC remain elusive, presenting significant challenges in its clinical management. Consequently, there is a pressing need for further research and comprehensive clinical studies to establish a more effective andpersonalize treatment strategy for this rare malignancy.

Prior to the introduction of immune checkpoint inhibitors (ICIs), this innovative class of drugs has revolutionized immunotherapeutic options for patients with ES-SCLC, significantly improving survival rates. The combination of ICI with the EP treatment regimen has become a cornerstone in the therapeutic strategy for patients with ES-SCLC (11, 13). Notably, the IMpower 133 regimen, which incorporates the PD-L1 antibody atezolizumab into platinum-based chemotherapy, has demonstrated enhanced overall survival (OS) compared to chemotherapy alone. In a similar vein, the CASPIAN regimen, which integrates the PD-L1 antibody durvalumab with chemotherapy, has also yielded improvements in overall survival (OS) (14). Consequently, adebrelimab is being considered as a potent therapeutic agent for tracheal SCC. Adebrelimab is a recombinant fully humanized IgG4 monoclonal antibody, exhibits high affinity and specificity for PD-L1. It was officially approved for cancer therapy by the China Drug Evaluation Center (CDE) in 2022 (15). The results of the multicenter phase III clinical trial of adebrelimab, employing the CAPSTONE-1 study design, have now solidified its role as a first-line treatment regimen for ES-SCLC (16). Specifically, this study enrolled a total of 462 patients with ES-SCLC, dividing them equally into two groups: the adebrelimab group, comprising 230 patients (50%) received adebrelimab in combination with chemotherapy, while the placebo group, also consisting of 232 patients (50%), received a placebo alongside chemotherapy. Patients in the adebrelimab group were treated with 4-6 cycles of carboplatin (area under the curve 5 mg/mL per minute, day 1) and etoposide (body surface area 100 mg/m^2^, days 1-3), augmented with adebrelimab (20 mg/kg, day 1). In contrast, the placebo group underwent 4-6 cycles of the same chemotherapy regimen, but with a placebo, over a treatment cycle of 21 days. Notably, the median overall survival in the adebrelimab group exhibited significant improvement compared to the placebo group. The most common grade 3 or 4 treatment-related adverse events were decreased neutrophil count, white blood cell count, platelet count, and hemoglobin level. The incidence of severe treatment-related adverse events was notably lower in the adebrelimab group. Importantly, there were two cases of treatment-related deaths reported in both the adebrelimab and placebo group among the four cases documented (17). Consequently, the addition of adebrelimab is observed to significantly enhance overall survival rate in patients with ES-SCLC, while maintaining an acceptable safety profile. These findings advocate for the implementation of this combination therapy as a novel first-line treatment option for patients with ES-SCLC.

This article presents a case of primary tracheal SCC in an 83-year-old male patient. The lung CT scan showed bronchiectasis and pulmonary emphysema, along with a lesion occupying the tracheal carina. Bronchoscopy revealed a neoplasm obstructing the lower part of the glottis, situated approximately 13 cm below the vocal cords. The histopathological analysis of the bronchial biopsy specimen, supplemented with immunohistochemical studies, confirmed the diagnosis of trachealSCC. This diagnosis was established based on the clinical data, morphological characteristics, and immunohistochemistry findings, indicating primary tracheal SCC without metastatic involvement. Tracheal SCC typically manifests as an intrabronchial mass. In this case, involving an 83-year-old patient with compromised baseline health and diagnosed with primary tracheal SCC(not secondary to other malignancies), we adhered to the treatment protocol from the CAPSTONE-1 study. Given the proven efficacy of immunotherapy combined with chemotherapy in treating ES-SCLC, we opted for a similar therapeutic regimen. Due to the patient’s advanced age and potential for chemotherapy resistance, a reduced dose of carboplatin was administered, alongside etoposide at 0.1g on days 1-5 and carboplatin 200mg on day 5, combined with adebrelimab 1.2g. Remarkably, after just two cycles of chemotherapy and immunotherapy, there was a significant reduction in the tracheal mass below the carina, and following six cycles, the mass completely disappeared without any severe adverse reactions. This combined approach of chemotherapy and immunotherapy demonstrated superior efficacy compared to chemotherapy plus radiotherapy, offering the patient a less distressing treatment course, maintaining overall well-being, and suggesting a novel therapeutic strategy for similar cases.

Bronchial carcinomas are relatively rare with small cell bronchial carcinoma being even more uncommon. Currently, the optimal treatment approach for such rare diseases remains unclear, The standard regimen typically involves platinum-based chemotherapy, commonly combining cisplatin and etoposide. This particular case of SCC offers a new perspective on potential treatment strategies. Taking into account the patient’s physical condition, the integration of immunotherapy with EP regimen can effectively enhance survival rates for patients with tracheal SCC, thereby presenting a novel therapeutic avenue.

## Data availability statement

The original contributions presented in the study are included in the article/**Supplementary Material**. Further inquiries can be directed to the corresponding author.

## Ethics statement

We asked the ethics committee and they said this type doesn’t require approval. The studies were conducted in accordance with the local legislation and institutional requirements. The human samples used in this study were acquired from a by- product of routine care or industry. Written informed consent to participate in this study was not required from the participants or the participants’ legal guardians/next of kin in accordance with the national legislation and the institutional requirements. Written informed consent was obtained from the individual(s) for the publication of any potentially identifiable images or data included in this article.

## Author contributions

YC: Writing – original draft. HZ: Writing – review & editing. DW: Resources, Writing – review & editing. YY: Resources, Writing – review & editing. JG: Resources, Writing – review & editing.
