# Supplementary figures and images for: Case report: A novel perspective on the treatment of primary tracheal small cell carcinoma: a patient’s experience with immuno-combined EP therapy and literature review
Source: Front Immunol. 2024 Jan 29;15:1356268. doi: 10.3389/fimmu.2024.1356268 (PMC10859462; doi:10.3389/fimmu.2024.1356268)

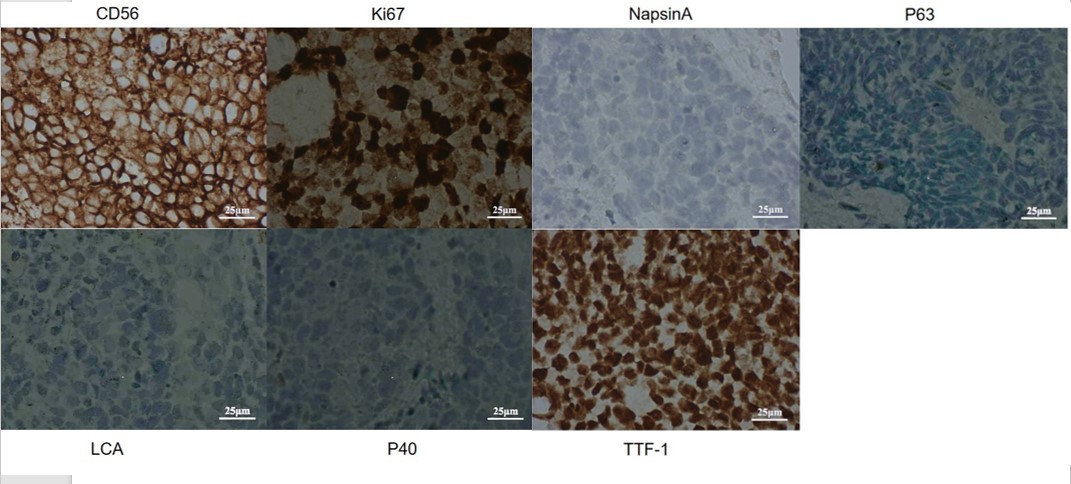

Supplement: Supplementary file 1 [file Image_1.jpg]
